# Acquired and Transmitted Multidrug Resistant Tuberculosis: The Role of Social Determinants

**DOI:** 10.1371/journal.pone.0146642

**Published:** 2016-01-14

**Authors:** Anna Odone, Roger Calderon, Mercedes C. Becerra, Zibiao Zhang, Carmen C. Contreras, Rosa Yataco, Jerome Galea, Leonid Lecca, Matthew H. Bonds, Carole D. Mitnick, Megan B. Murray

**Affiliations:** 1 Unit of Public Health, Department of Biomedical, Biotechnological and Translational Sciences, University of Parma, Parma, Italy; 2 Partners In Health / Socios En Salud, Boston, Massachusetts, United States of America and Lima, Peru; 3 Department of Global Health and Social Medicine, Harvard Medical School, Boston, Massachusetts, United States of America; 4 Division of Global Health Equity, Brigham and Women’s Hospital, Boston, Massachusetts, United States of America; 5 Department of Epidemiology, Harvard T.H. Chan School of Public Health, Boston, Massachusetts, United States of America; Seoul National University, REPUBLIC OF KOREA

## Abstract

Although risk factors for multi-drug resistant tuberculosis are known, few studies have differentiated between acquired and transmitted resistance. It is important to identify factors associated with these different mechanisms to optimize control measures. We conducted a prospective cohort study of index TB patients and their household contacts in Lima, Peru to identify risk factors associated with acquired and transmitted resistance, respectively. Patients with higher socioeconomic status (SES) had a 3-fold increased risk of transmitted resistance compared to those with lower SES when acquired resistance served as the baseline. Quality of housing mediated most of the impact of SES.

## Introduction

The new Post-2015 Global Tuberculosis Strategy identifies action on social determinants of tuberculosis as a main component of the bold policies and supportive systems required for TB control. In line with this Strategy, there is a general agreement that research is needed to assess and measure how social determinants affect TB risk [1].

At the population-level, improved socioeconomic conditions have been accompanied by a decline in the tuberculosis burden in industrialized countries over the past century. However, the association between socioeconomic status (SES) and TB is mediated by different proximal risk factors operating at different points in the natural history of TB [2,3]. The magnitude and direction of the association may therefore vary by setting, study population and the clinical features of the disease [3].

Scant data are available on the association between SES and multidrug-resistant tuberculosis (MDR-TB). Drug-resistant TB can result from two mechanisms: i) the selection for resistant bacteria in a patient undergoing treatment—defined as *acquired* (or *secondary*) resistance, or ii) infection with a drug resistant strain, defined as *transmitted* (or *primary*) resistance. Although previous studies have identified risk factors for MDR-TB [4,5], few studies have differentiated between risk factors for acquired and transmitted resistance or elucidated the role of SES in these two mechanisms.

Distinguishing between risk factors for acquired and those for transmitted MDR-TB may provide useful evidence to plan, implement, and evaluate targeted, preventive interventions in the context of TB control programs. Here, we analyze the relationship between individual and household-level SES and behavioral factors and *acquired* and *transmitted* MDR-TB in an urban South American setting.

## Methods

### Ethics Statement

The study was reviewed and approved by two IRBs. The first is the Research Ethics Committee of the National Institute of Health of Peru in Lima, Peru. The second is the Office of Human Research Administration at the Harvard School of Public Health.

Written informed consent was obtained from subjects older than 18 years old included in the study and from the guardians of subjects included in the study who were age 16–17 years.

### Study Population

We conducted a prospective cohort study on the epidemiology of MDR tuberculosis in urban Lima, Peru [6]. The primary objectives of the study were to measure the transmissibility of DR TB compared to drug-sensitive(DS) TB and to identify host and environmental factors associated with developing TB infection and disease. The study population comprised index TB patients >16 years diagnosed by the Peruvian National TB program in all outpatient public health centers of 23 urban districts and their household contacts. Individual written consent was sought from all subjects included in the study.

### Data

For each case, we collected data on demographic and SES characteristics, TB history and clinical status using clinical records and standardized questionnaires administered in-person by trained study workers. Sputum microscopy, mycobacterial cultures and first-line drug sensitivity tests were performed on samples collected for all patients. Mycobacterium tuberculosis(Mtb) strains were genotyped using 24-loci mycobacterial interspersed repeats (MIRU) and variable-number tandem repeats(VNTR) using standard methods [7]. Patients were started on treatment at the time of diagnosis and sputum smear. Cultures and drug resistance testing were repeated for patients with DS TB at 2 and 6 months and at 2, 6, 12, and 18 months for those who had MDR-TB.

### Definitions

We adopted a conservative approach to define *acquired* and *transmitted* resistance. In the past, a history of previous TB treatment in patients with DR TB has been used as a proxy for acquired resistance. However, ‘previously treated’ DR patients may include not only those who acquire resistance during previous treatment but also those who were primarily infected or re-infected with resistant strains. Along those lines, whether to consider ‘previous TB treatment’ as a proxy for acquired TB resistance has been debated in the literature and proved to fail to correctly identify subjects with acquired drug resistance [2–4]. To avoid potential misclassification, we classified a patient as having *acquired* DR only if 1)he/she was initially diagnosed with DS tuberculosis at study enrollment and later developed microbiologically confirmed resistance to any TB drug and if 2)his/her DS and subsequent DR Mtb isolates shared the same MIRU-based genotype. We considered a patient to harbor a *transmitted* MDR strain if his or her initial isolate was MDR **and** it shared a genotype with a strain from at least one other study participant **and** if all the other strains within a cluster of shared strains were multi-drug resistant. We considered study participants to be part of a cluster if the Mtb strain with which they were infected shared identical patterns for 24 loci VNTR-MIRU with one or more other isolated strains [8].

### Exposures of Interest and Principal Component Analysis

To assess the contribution of social determinants to DR risk, we considered individual-level proximal risk factors including smoking, drinking habits, and a history of imprisonment as well as distal determinants including education and household-level socioeconomic status (SES). All available household-level socioeconomic variables including housing quality water supply and sanitation data were included in a principal component analysis(PCA) to derive a measure of household-level SES(S1 Table)[9]. PCA is a common approach for generating wealth indices based on household asset information. PCA is a data reduction statistical technique that extracts a set of uncorrelated ‘principal components’ from a set of correlated variables, where each principal component is a weighted linear combination of the original variables. The weights (S1 Table) of the first principal component—which corresponds to the combination of the variables that explains the highest proportion of the variance—were used to generate a composite SES score as a continuous variable. The properties of the SES score were explored through histograms to verify the assumption of uniform distribution among the study population. The SES score was categorized into tertiles corresponding to relative “low, ” “middle, ” and “upper” SES. To explore the possible mediation pathway between SES and the defined outcomes, the variables used to compute the SES were also considered as separate exposures in sensitivity analyses.

### Analysis

We compared the distribution of exposures between subjects with transmitted and acquired DR TB reporting unadjusted and adjusted odds ratios(ORs); 95% confidence intervals(95%CI) were derived from univariate and multivariate logistic regression. Odds ratios for potential risk factors for transmitted resistance contrasted the odds of exposure in MDR-TB subjects with *transmitted* resistance with the odds of that exposure among subjects with *acquired* drug resistance. The final model was built on the basis of a conceptual framework derived from the literature describing the association between TB disease and SES, adapted to the study setting [10,11]. It included the covariates age, sex, education level, history of imprisonment, smoking, and drinking.

## Results

Between October 2009 and August 2012, we identified 3379 cases of culture-confirmed TB of which 411(12%) had at least one multi-drug resistant Mtb isolate at some point during the study. 29 subjects met our definition for *acquired* resistance and 61 for *transmitted* resistance. The remaining MDR cases could not be classified as having acquired or transmitted resistance based on these criteria.

Table 1 describes the characteristics of MDR patients by acquired, transmitted and unclassified status. Socioeconomic status was not differentially distributed between MDR-TB subjects included and excluded from our analysis (p = 0.7), nor were the other considered variables(Table 1).

**Table 1:** Socio-economic risk factors for acquired and transmitted resistance.

	ACQUIRED	TRANSMITTED	Unclassified MDR-TB cases	Unadjusted	Adjusted for age and sex	Final model	
	(n = 29)	(n = 61)	(n = 324)	OR (95%CI)	OR (95%CI)	OR (95%CI)	
	no.(%)	no.(%)	no.(%)				
**Education**							
Less than high school	13 (44.83)	23 (37.7)	119 (36.7)	1	1	1	
High school completed or more	16 (55.17)	38 (62.3)	205 (63.3)	1.34 (0.55–3.29)	1.18 (0.46–3.0)	0.63 (0.22–1.89)^a^	p = 0.41
**Prison**^a^							
No	28 (96.55)	53 (86.89)	291 (89.8)	1	1	1	
Yes	1 (3.45)	3 (4.92)	27 (8.3)	1.21 (0.54–2.72)	1.26 (0.47–3.36)	1.26 (0.45–3.51) ^a^	p = 0.6
missing	-	5 (8.2)	6 (1.9)				
**Smoking habit**							
Non-Smoker	27 (93.1)	56 (91.80)	301 (92.9)	1	1	1	
Smoker	2 (6.9)	2 (3.28)	16 (4.9)	1.58 (0.16–15.95)	2.46 (0.22–26.7)	1.27 (0.79–20.53) ^a^	p = 0.86
missing	-	3 (4.92)	7 (2.2)				
**Drinking habit**							
Non heavy drinker	26 (89.66)	49 (80.33)	265 (81.8)	1	1	1	
Heavy drinker	3 (10.34)	11 (18.03)	54 (16.7)	1.95 (0.5–7.6)	1.85 (0.46–7.45)	2.10 (0.39–10.90) ^a^	p = 0.38
missing	-	1 (1.64)	5 (1.5)				
**SES status**							
Low	10 (34.48)	18 (29.51)	117 (36.1)	1	1	1	
Medium	10 (34.48)	16 (26.23)	113 (34.9)	0.89 (0.29–2.68)	1.19 (0.36–3.87)	1.31 (0.37–4.64) ^a^	p = 0.08 (trend)
High	9 (31.03)	24 (39.34)	90 (27.8)	1.48 (0.50–4.40)	1.85 (0.59–5.76)	3.15 (0.81–12.18) ^a^	
missing	-	3 (4.92)	4 (1.2)				

^a^ adjusted for: age, sex, education, prison, smoking, drinking habit and SES status -

In the univariate analyses, subjects with transmitted resistance were younger, of higher socioeconomic status, more educated, more likely to have a history of incarceration or to be heavy drinkers than those with acquired resistance, although only age met the p = .05 criteria for statistical significance. After adjustment for age, sex and socioeconomic variables in the final model(Table 1), participants of “medium” and “high” SES were 1.31(95%CI:0.37–4.64) and 3.15(95%CI: 0.81–12.18) times as likely to have transmitted compared to acquired resistance (p for trend = 0.08). When we conducted a sensitivity analysis that considered housing alone among the SES variables, we found that participants with transmitted resistance were 3.18(95%CI: 1.10–9.58) more likely to have better housing than those with acquired resistance.

## Discussion

Our results suggest that in urban Lima higher socioeconomic status is associated with a nearly 3-fold increased risk of transmitted compared to acquired resistance and, conversely, that lower socioeconomic status is associated with an increased risk of acquired resistance. Quality of housing mediates most of the impact of SES. Despite this suggestive evidence, the results do not allow us to determine whether this effect is due to an increased risk of transmitted resistance in subjects of higher SES or an increased risk of acquired resistance in subjects of lower SES (although the distribution of SES status among the cases suggests the former) and we cannot quantify how much socioeconomic vulnerability contributes to MDR-TB burden through either mechanism.

One possible interpretation of our results is that better houses—where walls, roofs and windows are built with more solid materials—are less well ventilated and therefore increase the risk of within-household transmission. It is also possible that those of higher SES status work less and spend more time at home, thereby increasing their exposure within a household. Another possible pathway is that those with better houses may spend more time socializing indoors with increased risk of household crowding. On the other hand, wealthier people might have better access to healthcare, earlier access to TB diagnosis and supports permitting better treatment compliance, this reducing the risk of acquiring drug resistance. Case detection rates may also vary by SES strata and this might bias the results; although Peru has a strong public National TB Program that captures most cases, we cannot exclude this possibility in our study setting.

Our findings extend to transmitted MDR-TB the results of several studies from Africa which reported an unexpected association between measures of relative wealth and TB. Two consecutive cohorts in Malawi found that subjects living in higher quality houses were at higher risk of TB [10] while other measures of SES such as asset ownership were associated with diminished risk [10]. Similar findings have been reported from both high and low-incidence TB settings.

A large literature is available on how to measure socioeconomic status in relation to health outcomes [12]. Different socioeconomic measures capture different pathways of the association between socioeconomic status and tuberculosis. The rationale for using principal component analysis to derive a composite measure of relative socioeconomic status was to apply a recognized and increasingly used statistical technique and to obtain results easily comparable to other settings’ findings. We have previously applied the same analytical technique to explore the role of proximal risk factors in the relationship between TB and poverty in other settings [13].

The clustering definition we applied was supported by recent findings that demonstrated fully identical 24 loci VNTR typing result to be the most appropriate cluster definition in TB control practice [8].

Our work has some limitations. The conservative case definitions we adopted excluded most of the MDR-TB cases recruited in the cohort and we were therefore not able to assess the relative contribution of the two mechanisms to the MDR-TB burden in the study setting. Consequently, our small sample size did not allow us to identify any other than the most extreme associations and null findings should not be interpreted to suggest that other risk factors had no effect. Despite the limitations for statistical inference with small sample sizes, the p-value of .08 is weakly significant. Our strict definitions to differentiate between acquired and transmitted MDR-TB cases that combined both epidemiological and molecular features adds to the body of knowledge on the topic available in the literature and is one of the strengths of our study. A second limitation is that our findings do not allow us to derive conclusions generalizable to the entire Lima population; still, they allow us to suggest—on the basis of rigorously conducted analysis—that *acquired* and *transmitted* MDR-TB are associated with different risk factors that need to be taken into account when planning and implementing control programmes and interventions [14]. A third limitation, addressed above, is that we were unable to distinguish between the scenario in which higher SES facilitate transmission of MDR TB and that in which lower SES enabled acquisition of MDR TB.

To our knowledge this is the first study to assess the association between SES and MDR-TB distinguishing between *acquired* and *transmitted* resistance, especially using a prospective design and rigorous definitions that create more certainty about the classification of MDR TB into these categories. In particular, scant evidence is available on whether specific social determinants are proxies for exposure to patients with drug-resistant TB [15], or instead are associated with suboptimal care that selects for drug resistance. In the past, a history of previous TB treatment in drug resistant TB patients was used as a proxy for acquired resistance. However, because the ‘previously treated’ patient category can comprise patients primarily infected or re-infected with resistant strains, this approach may not reliably identify subjects with acquired drug resistance. Molecular epidemiologic approaches have sometime been used to differentiate between primary and secondary resistance; here, investigators have assumed that clustered MDR cases are those with primary resistance and those that are not clustered are due to acquired resistance. Like other molecular epidemiologic studies, however, the validity of this approach depends on the completeness of sampling and errors often results from convenience or small samples [16].

As stated in the recently released Global Tuberculosis Report—selected risk factors, including the emergence of DR and MDR tuberculosis poses a significant threat to global tuberculosis control strategies [17,18,19,20]. Acquired and transmitted resistance arise from two different mechanisms that are associated with different risk factors. We analyzed data from a large-scale prospective study and provide evidence suggesting that better living conditions might be associated with increased risk of MDR-TB transmission in urban settings. Health education programs should target specific subgroups of the population, promote good ventilation and prompt healthcare seeking and diagnosis so as to interrupt TB transmission. Further research is needed to confirm our findings. Our next step will be to geo-reference TB cases to explore the impact of ecological-level factors on MDR-TB transmission.

## Supporting Information

S1 Table Variables included in the principal component analysis (PCA) to derive socioeconomic status (SES). (DOCX)
