# Supplementary material for: Acquired and Transmitted Multidrug Resistant Tuberculosis: The Role of Social Determinants
Source: PLoS One. 2016 Jan 14;11(1):e0146642. doi: 10.1371/journal.pone.0146642 (PMC4713093; doi:10.1371/journal.pone.0146642)
Supplement: S1 Table — (DOCX) [file pone.0146642.s001.docx]

Table S1. Variables included in the principal component analysis (PCA) to derive socioeconomic status (SES)

| **Variable** | **Variable’s categories** | **Distribution in the study population**  **Mean (SD)** | **Weight in the *first* principal component** |
| --- | --- | --- | --- |
| Type of housing | Apartment or house | 0.88 (0.33) | 0.34 |
|  | worse than apartment/house  (Unit off alley way/ open Hall/ courtyard/Space not intended for human habitation/ Hut or Shack/Local provisional) |  |  |
| Total number of rooms in the house | More than one | 0.77 (0.42) | 0.27 |
|  | One |  |  |
| Exterior wall material | Brick or cement block | 0.87 (0.35) | 0.38 |
|  | worse than Brick or cement block  (Rock or ashlar with lime or cement/Adobe/Rammed earth/Sticks with mud/Rock with mud/wood/Matting) |  |  |
| Primary floor material | Parquet or polished wood/Asphalt or vinyl Tile/Tile or Teraza | 0.28 (0.45) | 0.25 |
|  | Cement or brick/Wooden Boards/earth |  |  |
| Primary roof material | Reinforced concrete | 0.66 (0.47) | 0.38 |
|  | Worse than reinforced concrete  (wood/Sheets of zinc/Tiles made with mud/Straw with mud/Straw/Thath, palm fronds) |  |  |
| Type of water supply | Public network inside home | 0.88 (0.32) | 0.47 |
|  | Worse than public network inside home  (public network outside home but inside building /public network spicket/ well in the house/ communal basin/cistern truck or similar) |  |  |
| Type of sanitation facilities | Public network inside home | 0.88 (0.32) | 0.48 |
|  | Worse than public network inside home  (no bathroom/River ditch or canal/blind well/septic tank/hom) |  |  |
| Type of lightening in the house | Electricity | 0.99 (0.11) | 0.09 |
|  | Worse than electricity  (no lighting/Oil or gas/ Candlelight/ Candlelight + Kerosene/ Electricity + Oil/Candlelight + Electricity/Electricity + Kerosene) |  |  |
